# Visual rigid laryngoscopy versus video laryngoscopy for endotracheal intubation in elderly patients: A randomized controlled trial

**DOI:** 10.1371/journal.pone.0309516

**Published:** 2024-10-22

**Authors:** Lijun Weng, Binmei Yu, Lan Ding, Menglu Shi, Tingjie Wang, Zengqiang Li, Weihuang Qiu, Xianzhong Lin, Bo Lin, Youguang Gao

**Affiliations:** 1 Department of Anesthesiology, Anesthesiology Research Institute, The First Affiliated Hospital, Fujian Medical University, Fuzhou, China; 2 Department of Anesthesiology, National Regional Medical Center, Binhai Campus of The First Affiliated Hospital, Fujian Medical University, Fuzhou, China; Sapienza University of Rome: Universita degli Studi di Roma La Sapienza, ITALY

## Abstract

**Objective:**

To assess the efficacy and safety of visual rigid laryngoscopy and video laryngoscopy and to provide clinical information for developing a more suitable intubation tool for elderly patients.

**Methods:**

In 75 consecutive elderly patients undergoing elective surgery in a single institution, tracheal intubation was randomly performed by 2 experienced anaesthesiologists using visual rigid laryngoscopy (Group I, n = 38) or video laryngoscopy (Group II, n = 37). The primary outcome was intubation time. Secondary outcomes were the first-attempt success rate of tracheal intubation, haemodynamic responses at 1, 3, and 5 min after intubation and the incidence of postoperative airway complications, including immediate complications and postoperative complaints.

**Results:**

The intubation times were 35.0 (30.0–41.5) s and 42.5 (38.0–51.3) s in Groups I and II, respectively (*P* < 0.001). The difference in direct complications between the two groups was statistically significant (*P* < 0.05). In contrast, there was no significant difference between the two groups regarding the follow-up of the main complaint 30 min and 24 h after tracheal extubation (*P* > 0.05). There was no difference in the intubation success rate between the 2 groups (*P* > 0.05). The haemodynamic responses at 1, 3, and 5 min after intubation were not significantly different (*P* > 0.05).

**Conclusion:**

Compared with that of video laryngoscopy, the intubation time of visual rigid laryngoscopy in elderly patients was shorter. At the same time, visual rigid laryngoscopy reduced the incidence of immediate complications. However, during endotracheal intubation, there was no significant difference in haemodynamics between the two groups.

**Clinical trial registration number:**

ChiCTR2100054174.

## Introduction

Degenerative changes in airway anatomy, such as the loss of cheek fat, shortening of the nail-chin distance, and changes in the head and neck joints, are common in elderly individuals; loose and missing teeth and even the presence of only one or two loose teeth near the incisors are also common [1, 2]. Structural changes in the airway may increase the incidence of dental injury, difficulty in mask ventilation [3, 4], and difficulty in tracheal intubation during the induction of anaesthesia [5, 6]. A correlation study indicated that in adult patients, the apnoea time with an oxygen saturation above 90% is only 1 min and a few seconds [7]. Elderly patients often have hypertension, diabetes mellitus, kidney disease and lung disease [8], are less tolerant to hypoxia and have reduced circulatory compensation [9]. Decreased vital organ function and reduced self-regulation in response to tracheal intubation stimulation are also common in elderly patients [10]. Moreover, various tracheal intubation tools have different structures, which may lead to different stress reactions [11] and intubation-related complications.

In recent years, video laryngoscope has been increasingly used for tracheal intubation in difficult airways. To date, many studies have confirmed that video laryngoscope is superior to direct laryngoscope in a variety of clinical situations, and the use of video laryngoscope can improve the efficiency and safety of tracheal intubation [12–15]. Visual rigid laryngoscopy has the advantage of easy operation [16, 17]. The low requirements for mouth opening and the "three lines in one" rule during visual rigid laryngoscopy intubation have significantly improved difficult airway management in patients with obesity, severely restricted mouth opening [18], and cervical spine disease [19], and especially in patients with cervical spine trauma [20]. Visual rigid laryngoscopy not only reduces the response to tracheal intubation [21] and significantly improves the success rate but also significantly reduces damage to the teeth, oral cavity and mucous membranes of peri-pharyngeal tissues during intubation.

There are numerous comparative studies between various laryngoscopy intubation techniques, in which both visual rigid laryngoscopy and video laryngoscopy are used as tracheal intubation tools in clinical anaesthesia. However, a comparison of these two intubation tools in elderly patients undergoing tracheal intubation has not been reported. A prospective randomized controlled study was conducted in order to compare visual rigid laryngoscopy with video laryngoscopy, as well as to provide clinical information to identify the most effective and safe laryngoscopy method.

## Materials and methods

### Trial design

This was a parallel-group, randomized, controlled trial.

### Participants, eligibility criteria, and settings

This study was performed after receiving approval from the ethical committee (MRCTA, ECFAH, of FMU [2021] 403), and the study protocol was registered at the Chinese Clinical Trial Registry (clinical trial registration number: ChiCTR2100054174) before patient enrolment. Each patient gave written informed consent before participation in the study. The study execute time was from December 14, 2021 to June 30, 2022. The study was carried out in the operating theatre of the First Affiliated Hospital of Fujian Medical University, and 75 patients aged 65 years and older with American Society of Anesthesiologists (ASA) physical status classifications of I–III who were undergoing elective surgery were included. Patients who had a history of difficult intubation or at least one predicted predictor of difficult intubation, a preoperative heart rate less than 50 or greater than 100 beats per minute, an arterial blood pressure less than 90/60 mmHg or greater than 180/100 mmHg, or throat diseases and those who refused to participate were excluded. The predictors of difficult intubation included Mallampati grades III and IV, a history of obstructive sleep apnoea, an interincisal distance (maximal mouth opening)<3 cm, restricted neck movement (<90°), a thyromental distance <6 cm, and a BMI >30 kg·m^−2^. In Mallampati class I: complete visualization of the palate, including hard and soft, uvula, and faucial pillars. In Mallampati class II: cranial part of faucial pillars, hard and soft palate, the entire uvula is visible. In Mallampati class III: visualization of the entire palate (hard and soft) and root of the uvula only. In Mallampati class IV: visualization of hard palate only. The thyromental distance, which is measured along a straight line from the thyroid cartilage prominence to the lower border of the mandibular mentum with full head extension.

### Interventions

As soon as the patient arrived in the operating room, all the above-mentioned airway measurements were recorded by a blinded independent observer. Sitting and neutral neck positions were used to measure interincisal distance, thyromental distance, restricted neck movement and the modified Mallampati classification, which categorizes patients based on pharyngeal structures. Following that, all patients received standard monitors (3-lead electrocardiograms, noninvasive blood pressure measurements, and pulse oximetry), and the radial artery was cannulated for continuous blood pressure monitoring.

Once adequate bag-mask ventilation was considered possible, a standardized induction of general anaesthesia was performed, with sufentanil 1.5 μg·kg^−1^ and propofol 1.5–2.5 mg·kg^−1^ followed by cisatracurium 0.15 mg·kg^−1^. Bag-mask ventilation was continued for 3 minutes with 100% oxygen. Tracheal intubation was performed by 1 of the 2 attending anaesthesiologists. The anesthesiologists who performed the tracheal intubation each performed at least 50 successful tracheal intubations using visual rigid laryngoscopy and video laryngoscopy.

**Fig 1:**
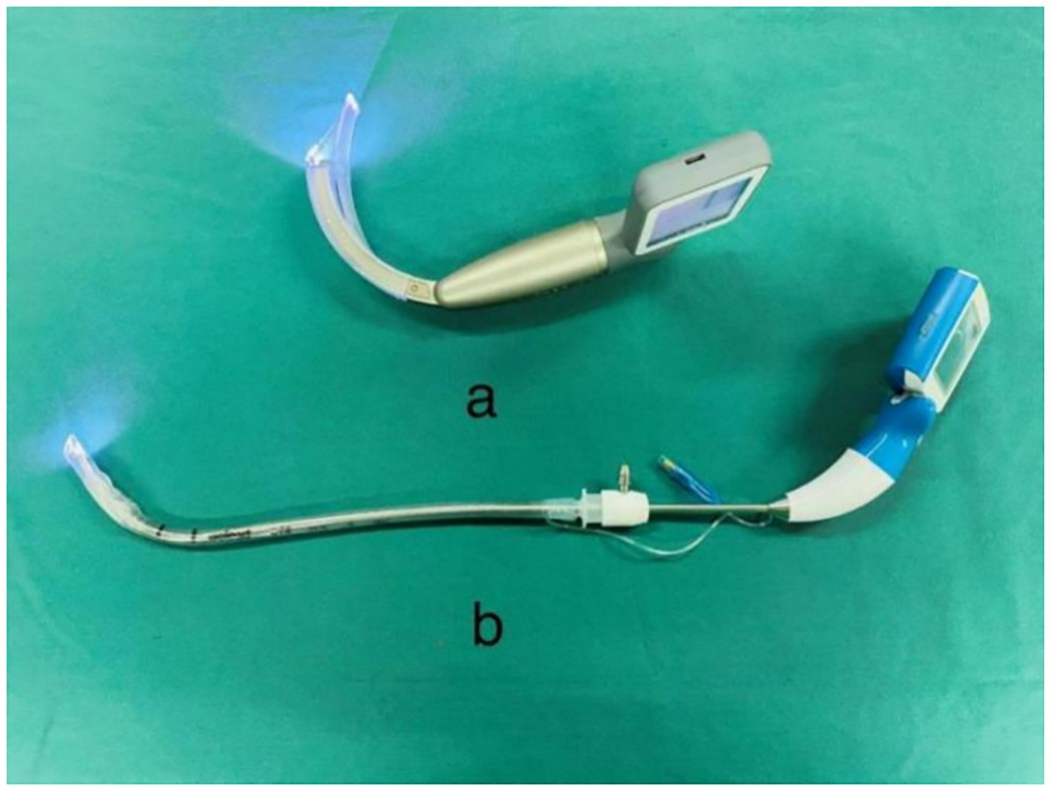
The picture of the laryngoscope. a: Visual laryngoscope, b: Visual rigid laryngoscope.

In Group I, a visual rigid laryngoscopy (Fig 1) was inserted with a preloaded endotracheal tube at the midline of the posterior pharynx. Once the vocal cords are seen around the epiglottis, the tip of the visual rigid laryngoscopy is advanced between the vocal cords and the tracheal tube is delivered into the trachea. In Group II, tracheal intubation was performed using a video laryngoscope (Fig 1). Both groups used reinforced tubes with an internal diameter of 7.0 mm for women and 7.5 mm for men.

### Outcomes (primary and secondary)

The primary outcome was intubation time. The intubation time was defined as the introduction of a visual rigid laryngoscope or video laryngoscope in the oral cavity until confirmation of proper positioning of the endotracheal tube by a positive capnography reading. Secondary outcomes were the first-attempt success rate of tracheal intubation, haemodynamic changes and the incidence of postoperative airway complications. End-tidal carbon dioxide monitoring using capnography was used to confirm that the tracheal intubation was successful. Mean arterial pressure and heart rate, as well as other hemodynamic changes, were measured before induction (T_1_), during intubation (T_2_), one minute after tracheal intubation (T_3_), three minutes after tracheal intubation (T_4_), and five minutes after tracheal intubation (T_5_). Immediate complications included lip or gum laceration, dental injury, the presence of blood in the oral cavity and blood stains on the laryngoscope. Further, a independent observer examined the patient’s airway after successful tracheal intubation for lip or gum laceration, dental injury, or bloody secretions. At 30 minutes and 24 hours following surgery, postoperative sore throat and hoarseness were assessed. A numerical rating scale (0, no pain; 10, the worst agony possible) was used to determine the degree of throat pain. From electronic medical records, information on postoperative neurological complications was gathered. These complications are indicated by newly appearing symptoms or a worsening neurological condition (paraesthesia, paresis, and paralysis) at hospital discharge. All of the outcomes were recorded by a blinded independent observer.

A tracheal intubation attempt was considered unsuccessful if it took longer than 180 seconds. After one minute of mask ventilation, the same anesthesiologist was given another try if the first one ended in failure. A maximum of three tries from the same anesthesiologist were permitted in each case. Intubation using a visual rigid laryngoscope in conjunction with a video laryngoscope was performed if all other attempts were unsuccessful. Blood staining on the laryngoscope was also assessed after intubation.

### Sample size calculation

The minimum sample size is estimated on the basis of intubation time. According to the pretrial results, the video laryngoscopy intubation time in 20 elderly patients was 46±10 s. Compared to the video laryngoscopy insertion time, a 15% reduction in the visual rigid laryngoscopy insertion time was estimated [22]. A total sample size of 34 people in each group was required based on the sample size calculation for an α set at 0.05 and a power at 1−β = 0.8. The “loss to follow-up rate” was anticipated to be 10%, and a final sample of 38 was determined for each group.

### Randomization (random number generation, allocatiaon concealment, implementation)

A computer-generated randomization number was used in sealed opaque envelopes to assign patients to either the visual rigid laryngoscopy (Group I) or video laryngoscopy (Group II) groups in a 1:1 ratio. As soon as anaesthesia was inducted and bag-mask ventilation was successfully achieved, the envelope was drawn in the presence of other staff immediately.

### Blinding

Two anesthetists, who were not informed about the use of the visual rigid laryngoscopy or video laryngoscopy until the patient arrives in the operating room, performed all intubations. An investigator who was unaware of the study’s existence recorded all the data.8.

### Statistical methods

All data were analysed using two-sample t-test power analysis [23, 24], and graphs were drawn using GraphPad Prism 8.0 (GraphPad Software Inc., San Diego, CA, USA). The results of quantitative data are presented either as median plus inter quartile ranges (for data with a non-Gaussian distribution) or mean ± standard deviation (SD) (for normally distributed data). Categorical data were summarised as the percentage of the total group. For quantitative data, differences in distribution between the two groups were evaluated using either the Wilcoxon/Mann–Whitney rank test (for data with nonGaussian distribution) or Student’s t-test (for normally distributed data). Comparison of different time points was performed by repeated measures ANOVA (for normally distributed data). Count data are expressed by rates, percentages or constituent ratios, and statistical analysis was carried out by chi square or Fisher’s exact tests. *P* < 0.05 was considered significant.

## Results

**Fig 2:**
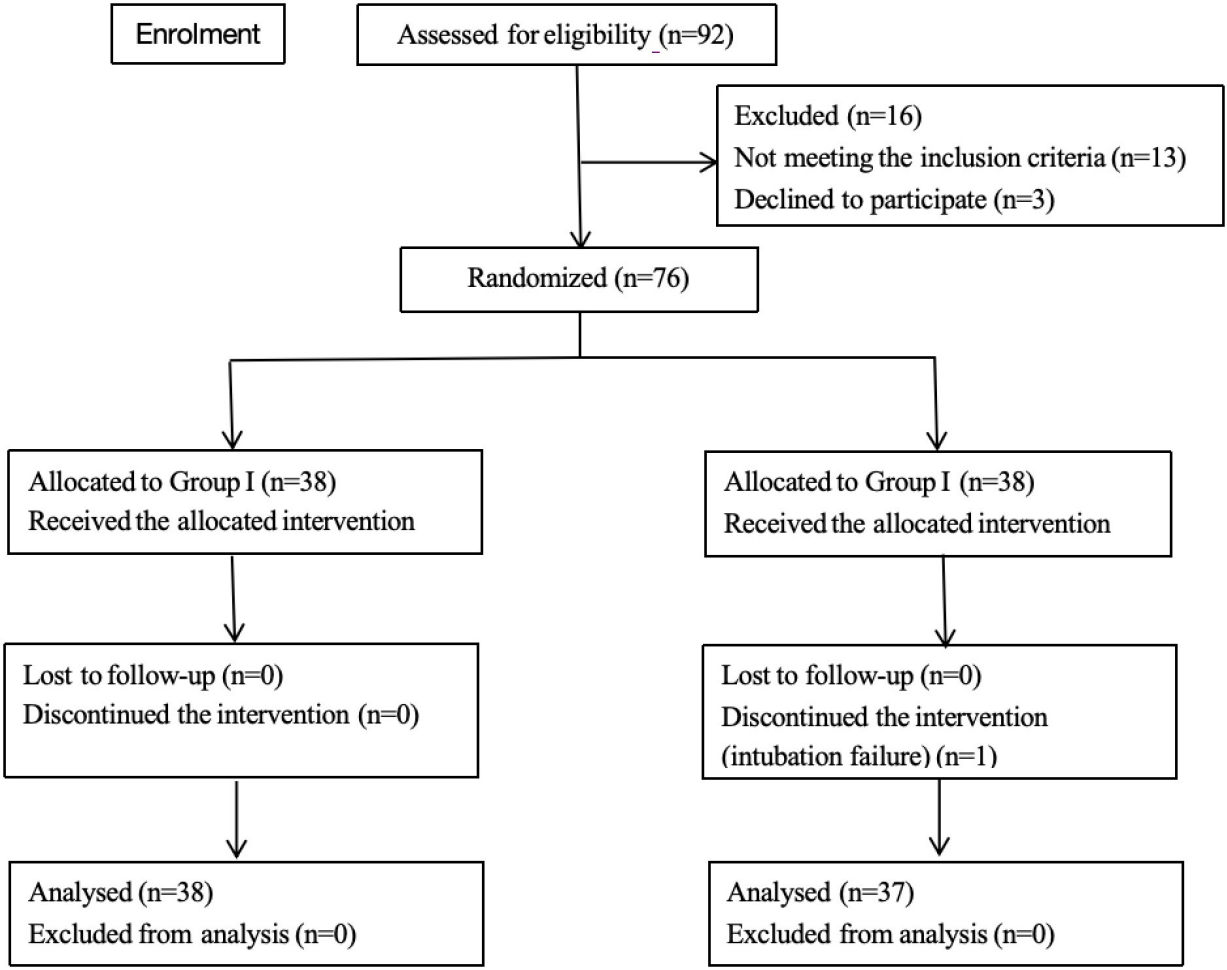
Consolidated Standards of Reporting Trials (CONSORT) flow diagram.

Among the 92 patients who fulfilled the inclusion criteria, 16 were excluded (11 for a history of difficult intubation or 1 or more predictors of difficult intubation, 2 for throat diseases, and 3 for refusal to participate) (Fig 2). One patient in Group II was excluded due to intubation failure, and 75 patients were evaluated.

There were no statistically significant differences between the groups in terms of demographic characteristics and airway-related variables (*P* > 0.05) (Table 1).

**Table 1:** Comparisons of demographic characteristics and airway-related variables between the 2 groups.

	Group I (n = 38)	Group II (n = 37)	*P* value
Age (y)	69.50 (67.00–73.25)	69.0 (67.00–75.50)	0.852
Sex			0.744
Male, n (%)	26 (68.40)	23 (64.90)	
Female, n (%)	12 (31.60)	13 (35.10)	
Height (cm)	167.50 (158.00–170.00)	163.00 (155.00–169.05)	0.432
Weight (kg)	61.05 ± 9.45	59.31 ± 10.23	0.447
Body mass index (kg/m^2^)	22.63 ± 2.66	22.33 ± 2.96	0.650
Thyromental distance (cm)	7.00 (6.50–8.00)	7.0 (6.15–7.75)	0.226
Interincisor distance (cm)	4.50 (4.00–5.50)	5.00 (4.50–5.75)	0.293
Neck circumference (cm)	37.1 ± 3.4	36.6 ± 3.7	0.461
Dental situation			0.873
toothless, n (%)	2 (5.30)	3 (8.10)	
missing incisors, n (%)	8 (21.10)	8 (18.90)	
fixed incisors, n (%)	28 (73.70)	28 (73.00)	
Modified Mallampati classification			0.24
I, n (%)	24 (63.20)	28 (75.70)	
II, n (%)	14 (36.80)	9 (39.10)	
ASA physical status			0.595
I, n (%)	3 (7.90)	2 (5.40)	
II, n (%)	30 (78.90)	27 (73.0)	
III, n (%)	5 (13.2)	8 (21.60)	
Duration of tracheal tube indwelling (min)	187.5 (138.75–240)	180 (100–227.5)	0.464
Hypertension (n, %)	18 (47.40)	17 (45.9)	0.902

Values are the mean ± standard deviation or the number (proportion). In Groups I and II, tracheal intubations were performed using the visual rigid laryngoscope and visual laryngoscope, respectively. Group I: Visual rigid laryngoscopy group. Group II: Video laryngoscopy group.

**Fig 3:**
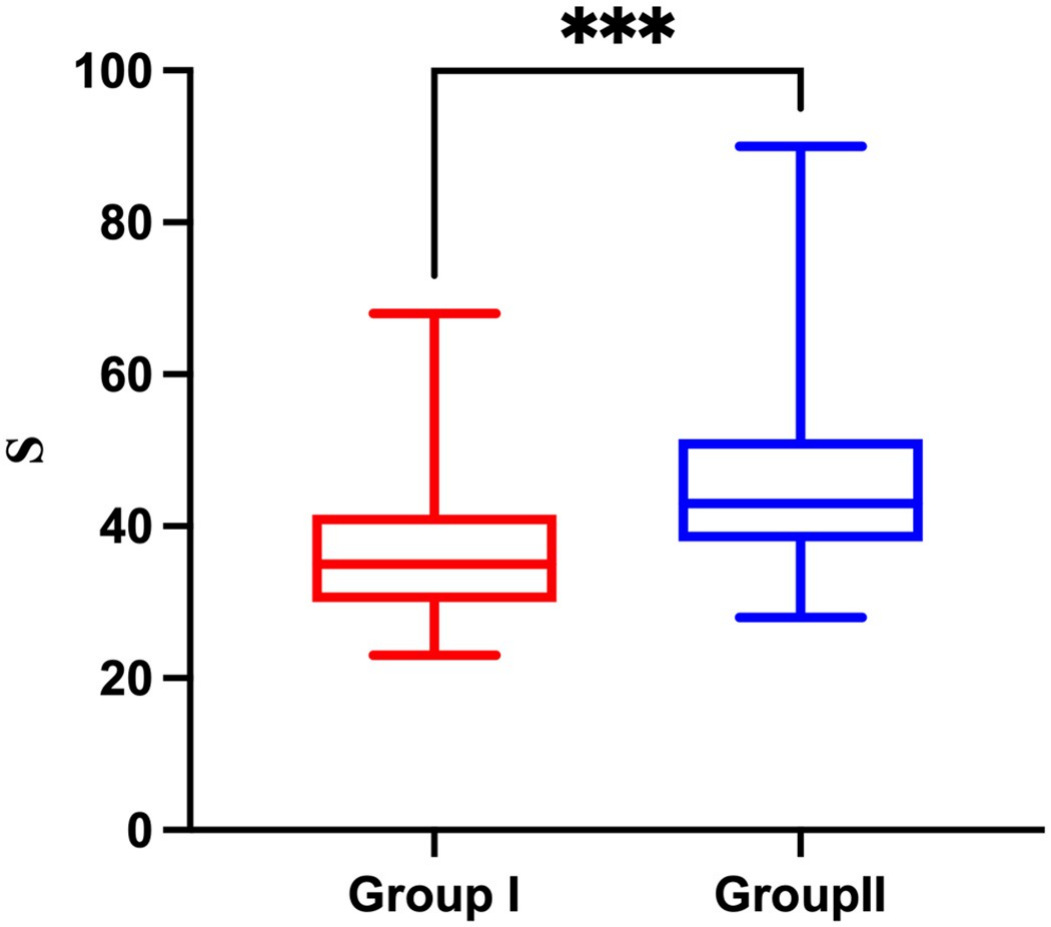
Comparison of intubation time between the two groups. The whiskers and line within each box show the minimum and maximum values, and the boxes themselves span the interquartile range. Group I: Visual rigid laryngoscopy group. Group II: Video laryngoscopy group. *P*<0.001.

The time of tracheal intubation in the visual rigid laryngoscopy group and video laryngoscopy group was 35.0 (30.0–41.5) s and 43 (38.0–51.5) s, respectively, and the intubation time of the visual rigid laryngoscopy group was shorter than that of the video laryngoscopy group (*P* < 0.001) (Fig 3).

The intubation was achieved in patients (75/76) in the group I and group II. The intubation success rate on the first attempt between Group I and Group II was similar. In the group I, 1/35 patient was intubated after the second attempt. In group II, 1/35 patient failed in tracheal intubation. There was no difference in the rate of successful intubation between the groups (*P* > 0.05) (Table 2).

**Table 2:** Comparisons of the rate of successful intubation between the 2 groups.

	Group I (n = 38)	Group II (n = 38)	*P* value
Success rate at each attempt			
1st attempt, n (%)	37 (97.36)	37 (97.36)	1.000
2nd attempt, n (%)	1 (2.63)	0 (0.00)	
3rd attempt, n (%)	0 (0.00)	0 (0.00)	
Overall, n (%)	38 (100)	37 (97.36)	0.987

Group I: Visual rigid laryngoscopy group. Group II: Video laryngoscopy group

**Fig 4:**
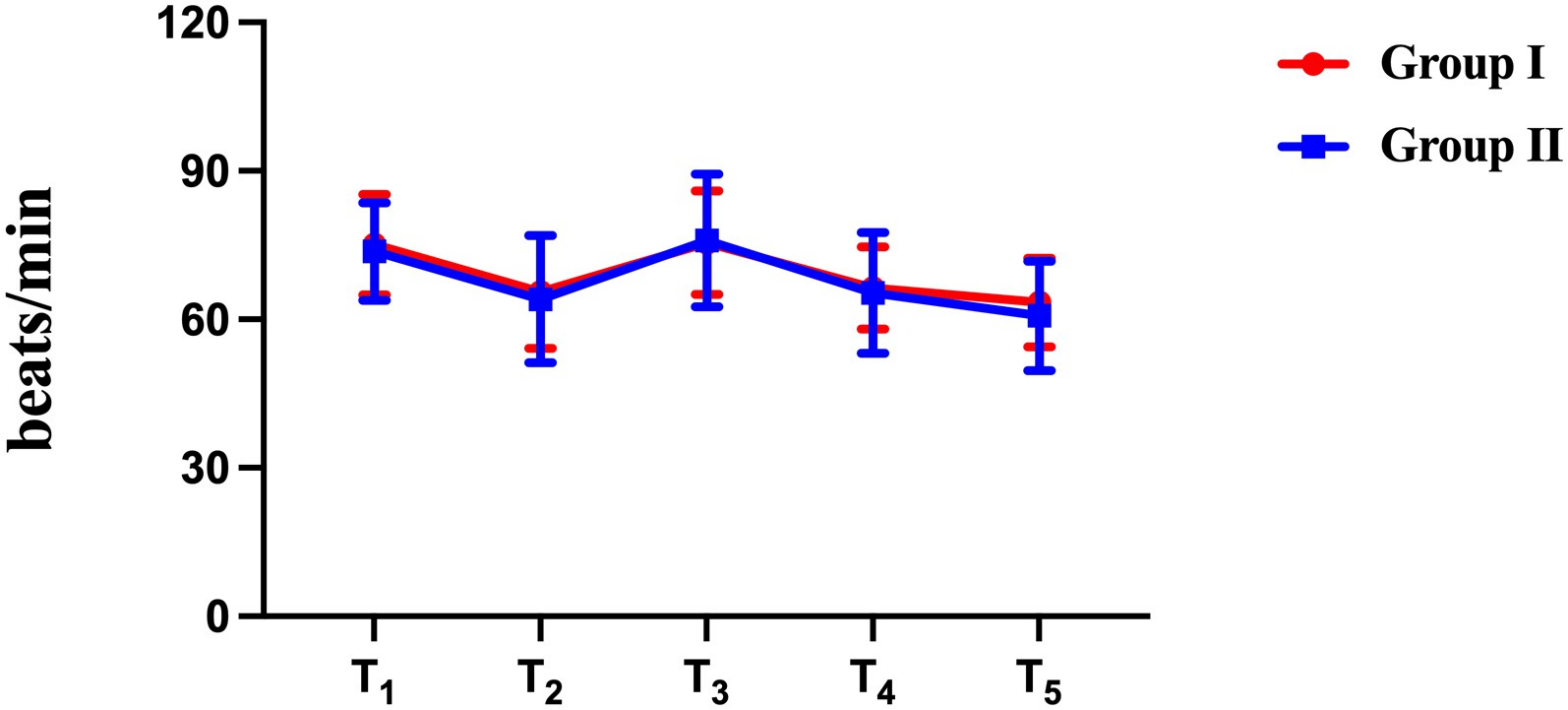
Changes in heart rate (HR) (mean) at different phases of intubation. There was no significant difference in the mean HR between the visual rigid laryngoscopy group and the video laryngoscopy group during different phases. *P* (T_1_) = 0.547; *P* (T_2_) = 0.601; *P* (T_3_) = 0.858; *P* (T_4_) = 0.682; *P* (T_5_) = 0.251. T_1_: Before the induction of anaesthesia; T_2_: Before tracheal intubation; T_3_: 1 min after tracheal intubation; T_4_: 3 min after tracheal intubation; T_5_: 5 min after tracheal intubation.

**Fig 5:**
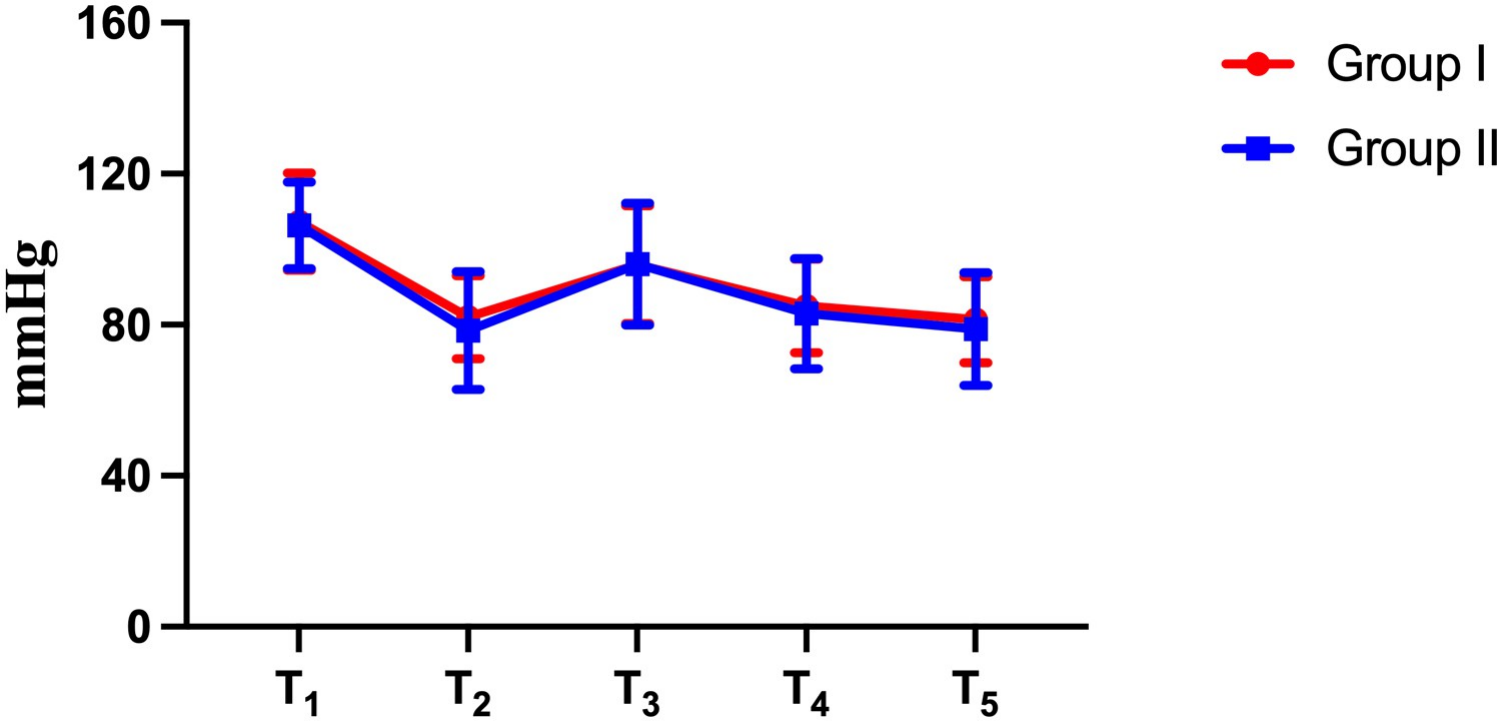
Changes in mean arterial pressure (MAP) (mean) at different phases of intubation. There was no significant difference in the mean MAP between the visual rigid laryngoscopy group and the video laryngoscopy group during different phases. *P* (T_1_) = 0.742; *P* (T_2_) = 0.255; *P* (T_3_) = 0.965; *P* (T_4_) = 0.521; *P* (T_5_) = 0.419. T_1_: Before the induction of anaesthesia; T_2_: Before tracheal intubation; T_3_: 1 min after tracheal intubation; T_4_: 3 min after tracheal intubation; T_5_: 5 min after tracheal intubation.

There were no statistically significant differences between the 2 studied groups regarding haemodynamics (*P* > 0.05) (Figs 4 and 5). Heart rate and mean arterial pressure increased during intubation in both groups. The increase in group II was more, but the increase was not much different from that in group I.

The difference in direct complications between the two groups was statistically significant (*P* < 0.05). In contrast, there was no significant difference between the two groups regarding the follow-up of the main complaint 30 min and 24 h after tracheal extubation (*P* > 0.05) (Table 3).

**Table 3:** Comparison of intubation-related complications between the two groups.

	Group I (n = 38)	Group II (n = 37)	*P* value
Direct complications, n (%)	1 (2.63)	7 (18.92)	0.022
lip injury, n (%)	0 (0.00)	1 (2.70)	0.493
laryngoscope with blood stains, n (%)	1 (2.63)	3 (8.10)	0.588
bleeding gums, n (%)	0 (0.00)	3 (8.10)	0.229
Postoperative complaints, n (%)	2 (5.26)	3 (8.10)	0.621
sore throat, n (%)	2 (5.26)	3 (8.10)	0.621
difficulty swallowing, n (%)	0 (0.00)	0 (0.00)	1.000
hoarseness, n (%)	0 (0.00)	0 (0.00)	1.000

## Discussion

This study showed that the tracheal intubation time using a visual rigid laryngoscope in elderly patients was significantly shorter than that using a video laryngoscope, and there were fewer intubation-related complications with the visual rigid laryngoscope than with the video laryngoscope. However, no significant differences were seen in the tracheal intubation success rate and fluctuations in haemodynamics between the two groups. There was one case of failed tracheal intubation using a video laryngoscope, and successful intubation was accomplished using a visual rigid laryngoscope combined with a video laryngoscope.

In this study, we found that the intubation time of the visual rigid laryngoscopy group was significantly shorter than that of the video laryngoscopy group. The reasons for this result could be attributed to the following aspects. First, the tracheal tube was placed on the rigid core before visual rigid laryngoscopy intubation, and the tracheal tube was delivered into the trachea as soon as the vocal cords were seen. For video laryngoscopy tracheal intubation, the vocal cords were first exposed with the laryngoscope, and then the tracheal tube was delivered into the trachea in two steps, which took a relatively longer time. Second, elderly patients have reduced head and neck mobility, loose or absent incisors, and reduced mouth opening. Compared with video laryngoscopy, visual rigid laryngoscopy requires less mouth opening and head and neck mobility, which makes it more suitable for elderly patients and completing intubation faster. Interestingly, it was shown that a decrease in the nail-chin distance, a predictor of intubation, predicted a shorter intubation time with visual rigid laryngoscopy [25]. Finally, the length of intubation time was also correlated with the operator’s proficiency in using a visual rigid laryngoscope and video laryngoscope.

The longer intubation time using visual rigid laryngoscopes in this study was mainly due to the smaller exposure of the pharyngeal space and the difficulty in identifying pharyngeal structures and bypassing the epiglottis during operation for patients with a narrow pharyngeal lumen and long epiglottis. If the laryngeal anatomy is not understood, it is impossible to locate the position of the glottis accurately and quickly. When using visual rigid laryngoscopy for tracheal intubation, it is crucial to establish the initial view, and a certain depth of intubation needs to be maintained. If unrecognizable pink tissue is encountered, it is appropriate to withdraw, return to the initial view, and use the uvula or epiglottis as a positioning marker for appropriate adjustment. In patients in whom bypassing the epiglottis is difficult, the posterior mandibular gap can be improved by lifting the jaw and tongue and extending through the neck [26].

The reduction in intubation time may not be clinically significant in healthy young adults, but it is of some significance in older patients. On the one hand, elderly people undergo many anatomical, physiopathological, and cognitive changes that affect different components of airway management: Intubation, ventilation, oxygenation, and the risk of aspiration [2]. On the other hand, as the duration of laryngoscopy use increases, the magnitude of the resulting stress response is greater [27]. The elevation in arterial pressure typically starts within five seconds of laryngoscopy, peaks at 1–2 min and returns to control levels within 5 min [28]. Elderly patients with reduced vascular elasticity and self-regulation may experience stress reactions, such as myocardial hypoxia and hypertension, as the duration of intubation and haemodynamic fluctuations increase.

There was no significant difference in the success rate of tracheal intubation between the two groups. The main reason for the failure of the first insertion of the visual rigid laryngoscope in this study was poor visualization of the larynx due to contamination of the lens by secretions. For a trained operator, Abdullah et al founded no difference in success for tracheal intubation with either the Bonfils or the McCoy laryngoscope [29]. And in Abdullah’s study, secretions also can limit the usefulness of a fiberoptic scope.

The failure of video laryngoscopy tracheal intubation in one case in this study was mainly due to an unanticipated difficulty in intubation due to the inability to provoke the epiglottis, resulting in difficulty exposing of the vocal cords. Subsequently, the intubators performed successful tracheal intubation using a visual rigid laryngoscope combined with a video laryngoscope. Because the combined use of the laryngoscopes increases their functionality, their respective advantages are realized [30]. First, the video laryngoscope provides increased exposure and the best possible view of the oral cavity and pharynx, and there is sufficient space to insert a visual rigid laryngoscope with a tracheal tube next to the video laryngoscope. Second, the rigid core structure of the visual rigid laryngoscope and the video lens at the front of the core help better guide the catheter over the epiglottis and into the vocal cords, improving the intubation view and increasing the intubation success rate. A study [31] used video laryngoscopy in combination with the Bonfils fiberscope to intubate 38 patients with an anticipated difficult airway. Thirty-seven of these patients were intubated successfully, and 33 had improved Cormack & Lehane scores. The combined use of visual rigid laryngoscopy and video laryngoscopy should provide an effective alternative for anaesthesiologists to perform tracheal intubation in patients with difficult airways.

This study showed no significant differences in haemodynamic fluctuations between tracheal intubation using visual rigid laryngoscopy and that using video laryngoscopy. The stress reaction caused by tracheal intubation is mainly due to two reasons [27]. First, the neural reflexes are elicited by the stimulation of the pharyngeal tissues and the voice box during the insertion and lifting of the laryngoscope. Second, the trachea is stimulated after insertion of the tracheal tube. Direct tracheal stimulation appears to be the main cause of the haemodynamic changes associated with tracheal intubation [32]. The haemodynamic response to tracheal intubation with the Bonfils fiberscope was also comparable to that of C-MAC video laryngoscopy in 50 patients scheduled for elective surgery in the study by Ezhar et al [21].

In this study, the incidence of direct complications related to intubation was 18.92% in the video laryngoscopy group compared with only 2.63% in the visual rigid laryngoscopy group. In addition, no significant difference was seen in the incidence of postoperative complaints, such as sore throat and hoarseness, between the two groups of elderly patients. Laryngoscopes are constructed differently and have very different forces [33, 34]. Although the force during intubation was not monitored in this trial, the visual rigid laryngoscope is lightweight, has a small tube diameter, and requires less mouth opening and head and neck mobility. In this trial, no dental injuries were observed during visual rigid laryngoscopy intubation, which shows its advantages.

This study had several limitations. First, the results of this study are not equally generalizable to operators who are not familiar with either device. Second, the use of tracheal intubation equipment in this study may differ from the use of other types of video laryngoscopes and visual rigid laryngoscopes. Third, the two intubation techniques are significantly different. Therefore, only patients and postoperative followers could be blinded, not the tracheal intubation operator, tracheal intubation observer, and data collector. Fourth, this study excludes patients with parameters predictive of difficult intubation, therefore it cannot refer to the context of difficult airway management. Finally, although the elderly patients were randomized in this study and the number of patients with hypertension in the two groups did not differ significantly, treatment with different antihypertensive drugs may have different effects on the haemodynamic response during tracheal intubation.

In conclusion, the time to tracheal intubation was reduced with visual rigid laryngoscopy compared with video laryngoscopy in elderly patients. Immediate complications during tracheal intubation were reduced with visual rigid laryngoscopy compared to video laryngoscopy. Visual rigid laryngoscope is more suitable than video laryngoscope for endotracheal intubation in the elderly. However, there was no significant difference in the comparison of haemodynamics during tracheal intubation between the two groups. At present, the conclusion is based on a random sample of the Chinese population, and samples from other regions can be collected in the future to increase the sample size and determine the effectiveness of the intervention. The choice to use either device should be based on the patient’s basic condition, available assistive devices, and the operator’s personal experience.

## Supporting information

S1 Dataset The relevant data. (XLSX)

S2 Dataset The specific pretrial data. (XLSX)

S1 Protocol Trial study protocol. (DOCX)

S1 Checklist CONSORT 2010 checklist of information to include when reporting a randomised trial*. (DOC)
