# Supplementary material for: Visual rigid laryngoscopy versus video laryngoscopy for endotracheal intubation in elderly patients: A randomized controlled trial
Source: PLoS One. 2024 Oct 22;19(10):e0309516. doi: 10.1371/journal.pone.0309516 (PMC11495622; doi:10.1371/journal.pone.0309516)
Supplement: S1 Protocol — (DOCX) [file pone.0309516.s003.docx]

**Visual rigid laryngoscopy versus video laryngoscopy for endotracheal intubation in elderly patients: A randomized controlled trial**

1. **Research background and purpose**

Tracheal intubation causes adverse hemodynamic responses, including increased arterial blood pressure and heart rate. Because the endotrachealin tubation reaction is induced after general anesthesia, the endotracheal in tubation operation stimulates the oropharyngeal and laryngeal structures rich in sympathetic nerve, and the terminal hormone cortisol and catabolic hormone of pituitary gland and adrenal cortex in the blood, such as the sharp increase of catecholamine concentration causes a series of cardiovascular reactions.

Hemodynamic responses can aggravate certain risks, such as myocardial infarction and stroke, especially in elderly patients. Elderly patients often have hypertension, diabetes mellitus, kidney disease and lung disease, are less tolerant to hypoxia and have reduced circulatory compensation. For general anesthesia induction in elderly patients, not only appropriate anesthesia induction drugs, but also small stimulating in tubation should be selected to minimize the time of hypoxia, reduce hemodynamic fluctuations, and increase the success rate of in tubation. In addition, elderly patients with loose teeth, incomplete common, and even the upper and lower incisors only one tooth. Common laryngoscopic endotracheal in tubation, it is difficult to avoid not damaging the teeth, and even can lead to the original loose teeth loss or bleeding of the gums. Therefore, the requirements for in tubation equipment is put forward.

Video laryngoscope is a video-type in tubation system with an external display device. It uses the camera at the front end of the laryngoscope to present the glottis and the surrounding tissue structure to the external display device in real time. The intubation personnel can quickly and accurately insert the endotracheal tube into the appropriate location according to the real-time image on the display device. Compared with the traditional way of direct laryngoscopy, video laryngoscope can clearly show the tissues near the larynx and the glottis, which can help in tubation personnel reduce the damage caused by blind endotracheal in tubation and reduce the occurrence of complications related to the tongue, throat and trachea after surgery. Visual rigid laryngoscope its front bending is "J" shape, endotracheal tube directly on the mirror, under the direct push tube into the trachea, with easy to operate, low requirement for mouth, small damage, high success rate, during in tubation through the glottis structure and tracheal tube tip display, significantly improve the glottis show classification, can reduce the glottis show classification above grade I. Visual rigid laryngoscope does not need olfactory position, and the patient's mouth opening, tooth condition and mouth and throat space are low, avoid the exposure of the glottis, lips and teeth, reduce damage, and effectively reduce the contact degree and intensity of the mouth and throat, reduce the stimulation of the root of the tongue and throat. Both are currently commonly used endotracheal in tubation tools in clinical anesthesia. However, the comparison of these two endotracheal in tubation tools in elderly patients undergoing orotracheal in tubation has not been reported yet.

At present, there are more and more types of in tubation tools. This study aims to compare the application of visual rigid laryngoscope and video laryngoscope in elderly patients with orotracheal in tubation, and to seek more suitable in tubation tools to shorten the time of endotracheal in tubation, increase the success rate of in tubation, reduce the occurrence of complications and maintain hemodynamic stability.

1. **Research content**

A computer-generated randomization number was used in sealed opaque envelopes to assign patients to either the visual rigid laryngoscopy (Group I) or video laryngoscopy (Group II) groups. Elderly patients undergoing elective surgery were selected and patients were randomized by random number chart and selected by video laryngoscopy. The two groups were evaluated the glottic exposure during in tubation, and recorded the in tubation time, in tubation conditions, incidence of difficult in tubation, success rate of in tubation and direct complications

associated with in tubation. Then, we recorded the effects of the hemodynamics between the two groups. At 30 minutes and 24 hours following surgery, postoperative sore throat and hoarseness were assessed.

1. **Research protocol**

The study was carried out in the operating theatre of the First Affiliated Hospital of Fujian Medical University, and 75 patients aged 65 years and older with American Society of Anesthesiologists (ASA) physical status classifications of I–III who were undergoing elective surgery were included. Patients who had a history of difficult intubation or at least one predicted predictor of difficult intubation, a preoperative heart rate less than 50 or greater than 100 beats per minute, an arterial blood pressure less than 90/60 mmHg or greater than 180/100 mmHg, or throat diseases and those who refused to participate were excluded. The predictors of difficult intubation included Mallampati grades III and IV, a history of obstructive sleep apnoea, an interincisal distance <3 cm, restricted neck movement (<90°), a thyromental distance <60 cm, and a BMI >30 kg·m−2.

As soon as the patient arrived in the operating room, all the above-mentioned airway measurements were recorded. Sitting and neutral neck positions were used to measure the modified Mallampati classification, which categorizes patients based on pharyngeal structures. Following that, all patients received standard monitors (3-lead electrocardiograms, noninvasive blood pressure measurements, and pulse oximetry), and the radial artery was cannulated for continuous blood pressure monitoring.

Once adequate bag-mask ventilation was considered possible, a standardized induction of general anaesthesia was performed, with sufentanil 1.5 μg·kg−1 and propofol 1.5-2.5 mg·kg−1 followed by cisatracurium 0.15 mg·kg−1. Bag-mask ventilation was continued for 3 minutes with 100% oxygen. Tracheal intubation was performed by 1 of the 2 attending anaesthesiologists. The anesthesiologists who performed the tracheal intubation each performed at least 50 successful tracheal intubations using visual rigid laryngoscopy and video laryngoscopy.

In Group I, a visual rigid laryngoscopy was inserted with a preloaded endotracheal tube at the midline of the posterior pharynx. Once the vocal cords are seen around the epiglottis, the tip of the visual rigid laryngoscopy is advanced between the vocal cords and the tracheal tube is delivered into the trachea. In Group II, tracheal intubation was performed using a video laryngoscope. Both groups used reinforced tubes with an internal diameter of 7.0 mm for women and 7.5 mm for men.

The primary outcome was intubation time. The intubation time was defined as the introduction of a visual rigid laryngoscope or video laryngoscope in the oral cavity until confirmation of proper positioning of the endotracheal tube by a positive capnography reading. Secondary outcomes were the first-attempt success rate of tracheal intubation, haemodynamic changes and the incidence of postoperative airway complications. End-tidal carbon dioxide monitoring using capnography was used to confirm that the tracheal intubation was successful. Mean arterial pressure and heart rate, as well as other hemodynamic changes, were measured before induction (T1), during intubation (T2), one minute after tracheal intubation (T3), three minutes after tracheal intubation (T4), and five minutes after tracheal intubation (T5). Following intubation, the presence of blood in the oral cavity and blood stains on the laryngoscope were assessed. At 30 minutes and 24 hours following surgery, postoperative sore throat and hoarseness were assessed. A numerical rating scale (0, no pain; 10, the worst agony possible) was used to determine the degree of throat pain. From electronic medical records, information on postoperative neurological complications was gathered. These complications are indicated by newly appearing symptoms or a worsening neurological condition (paraesthesia, paresis, and paralysis) at hospital discharge. An investigator who was unaware of the study's existence recorded all of the data.

1. **potential difficulties and solutions encountered in the research process**
2. If endotracheal in tubation with video laryngoscope and video rigid

laryngoscope is difficult, we will jointly use video laryngoscope and video rigid laryngoscope for in tubation or use laryngeal mask and other airway emergency devices to ensure the safety of patients.

(2) These patients were excluded due to various reasons, such as temporary withdrawal of patients from the experiment, incomplete collection of information on patients' main indicators, or loss of follow-up after surgery

**5. Methods of obtaining materials and confidentiality measures**

Materials were mainly obtained through inpatient cases, examination and test reports, surgical anesthesia records, laryngoscopy images, etc. The personal information of participants in the study will be kept strictly confidential and personal information will not be disclosed to the outside world. When necessary, other than the hospital ethics committee and relevant researchers can review the subject's data, no one will have access to relevant personal information.
